# Osteopontin N-Terminal Function in an Abdominal Aortic Aneurysm From Apolipoprotein E-Deficient Mice

**DOI:** 10.3389/fcell.2021.681790

**Published:** 2021-08-12

**Authors:** Hongyang Liu, Ying Zhang, Wei Song, Yancui Sun, Yinong Jiang

**Affiliations:** ^1^Department of Heart Intensive Care Unit, The First Affiliated Hospital of Dalian Medical University, Dalian, China; ^2^Department of Cardiology, The First Affiliated Hospital of Dalian Medical University, Dalian, China

**Keywords:** osteopontin N-terminal, abdominal aortic aneurysm, pyroptosis, ApoE-/- mice, inflammation

## Abstract

The cleavage of osteopontin (OPN) by thrombin results in an N-terminal fragment (OPN-N), which exposes a cryptic integrin-binding motif that promotes the adherence of cells, and plays a proinflammatory role. However, the effect of OPN-N on abdominal aortic aneurysm (AAA) remains unknown. The aim of this study was to investigate the expression of OPN-N in aortic tissue samples obtained from patients, who underwent acute aortic dissection (AD), and normal aorta, effect of OPN-N on angiotensin (Ang) II-induced AAA in mice, and relationship between OPN-N and pyroptosis-related inflammatory factors *in vitro*. Hematoxylin and eosin staining was conducted to detect histological changes. Next, we detected the expression of the OPN-N protein. Additionally, ApoE−/− mice were divided into four groups: control, control + M5Ab (to block the OPN-N function in mice), Ang II, and Ang II + M5Ab. All mice were euthanized after a 28-day infusion and whole aortas, including thoracic and abdominal aortas, were collected for morphological and histological analysis of the AAA. The OPN-N protein expression was higher in patients with AD than in normal individuals, while histological changes in the aortas of Ang II mice were suppressed in Ang II + M5Ab mice. The expression of OPN-N, NOD-, LRR-, and pyrin domain-containing protein 3, pro-Caspase-1, ASC, Gasdermin-d, interleukin (IL)-18, IL-1β, matrix metalloproteinase (MMP) 2, and MMP9 was lower in the Ang II + M5Ab group than in the Ang II group. The gene expression of monocyte chemoattractant protein-1, IL-6, and tumor necrosis factor-α was suppressed in the aortic tissues of the Ang II + M5Ab group compared with the Ang II group. Moreover, the expression of α-smooth muscle actin was lower in the Ang II group than in the Ang II + M5Ab group. *In vitro* results showed that the increase in the expression of pyroptosis-related inflammatory factors induced by OPN was mediated through the nuclear factor (NF)-κB pathway. In conclusion, OPN-N promotes AAA by increasing the expression of pyroptosis-related inflammatory factors through the NF-κB pathway, inflammation, and extracellular matrix degradation. These results highlight the potential of OPN-N as a new therapeutic target to prevent AAA expansion.

## Introduction

Abdominal aortic aneurysm (AAA) is a cardiovascular disease characterized by aortic dilation that exceeds the normal diameter by 50% (Nordon et al., 2011). Older age, male sex, smoking, hyperlipidemia, and atherosclerosis are risk factors associated with AAA (Ahmed et al., 2016; Chaikof et al., 2018). The AAA morbidity is 4–8% in elderly patients (>65 years), which is expected to increase with the aging of population (Howard et al., 2015; Maguire et al., 2019). Despite progress in surgical treatment, strategies to predict AAA rupture or delay its progress remain lacking.

The pathophysiology of AAA is complex and includes a significant aortic tissue inflammatory response (Hellenthal et al., 2009; Raffort et al., 2017). Previous studies have shown that inflammatory cells secrete various inflammatory factors, including cytokines, chemokines, reactive oxygen species, and immunoglobulins (Ihara et al., 1999; Vanderlaan and Reardon, 2005; Liu et al., 2019). This causes macrophages and lymphocytes to infiltrate the arterial wall, which results in the release of excess matrix metalloproteinase (MMP), degradation of elastin in the aortic wall, phenotype switch and loss of vascular smooth muscle cells (VSMCs), and compensatory collagen deposition (Raffort et al., 2017; Maguire et al., 2019; Wang S. et al., 2020). Additionally, an animal study has shown that the inhibition of inflammation and MMP activity reduces aneurysm (Shimizu et al., 2004). Pyroptosis is an inflammatory cell death process induced by the activation of Caspase-1, which is activated by the NLRP3 inflammasome (Miao et al., 2010) which in turn has been shown to play an important role during the initial inflammatory response to AAA formation and is a potential therapeutic target against AAA (Sun et al., 2015; Usui et al., 2015; Takahashi, 2021). Canonical inflammasomes consists of at least three proteins: the adapter molecule ASC (apoptosis-associated speck-like protein containing CARD, PYCARD), the effector of pro-Caspase-1, the sensor protein of pattern-recognition reptor (PRR). To date, the NLRP3 inflammasome-forming PRR which is well characterized in the inflammatory diseases. The fully active Caspase-1 then cleaves pro-IL-1β and pro-IL-18 into their biologically active forms as well as Gasdermin-d (GSDMD), which is not only necessary for the secretion of cytosolic contents, but also induce lytic cell death termed “pyrotosis” (Takahashi, 2021).

Osteopontin (OPN) is not only a secreted matricellular cytokine, but also a circulating pro-inflammatory cytokine, which can induce cell adhesion, migration, and activation of inflammatory pathways. Chronic increase of OPN expression is associated with an increased risk of major adverse cardiovascular events (Wang et al., 2018; Lok and Lyle, 2019). Previous studies have reported OPN to be the driving force behind the formation and growth of AAA (Bruemmer et al., 2003; Zheng et al., 2012; Wang et al., 2018). During inflammatory cell infiltration, OPN can be cleaved by thrombin into two fragments at ^168^R/S^169^ site, one of which is the N-terminal fragment of OPN (OPN-N) which exposes a cryptic integrin binding motif, ^162^SVVYGLR^168^ (SLAYGLR in mice), and binds to α4β1 and α9β1 integrins, thereby exerting a strong pro-inflammatory effect (Yamamoto et al., 2003; Yamaguchi et al., 2013). In mice, the OPN-N has been implicated in inflammation and tissue injury. For instance, Boggio et al. (2016) has demonstrated that OPN-N plays a key role in multiple sclerosis by increasing the production of interleukin (IL)-17 and IL-6. Additionally, OPN-N accelerates cardiac damage in a rat model of ischemic cardiomyopathy (López et al., 2013; Uchinaka et al., 2015), while another study has shown that OPN-N was strongly associated with inflammation and instability of carotid atherosclerotic plaques in patients with hypertension (Wolak et al., 2013). A polyclonal antibody, M5Ab, against a synthetic peptide (a cryptic epitope of OPN exposed by thrombin cleavage, VDVPNGRGDSLAYGLRS, M5 peptide) has been shown to block OPN-N function in mice (Diao et al., 2008; Cui et al., 2019).

However, the effect of OPN-N on AAA remains unknown. Therefore, we investigated the expression of OPN-N in aortic tissue samples of patients, used M5Ab to block the function of OPN-N and investigated the role of OPN-N in mice with angiotensin II (Ang II)-induced AAA, and evaluated the association between OPN-N and pyroptosis-related inflammatory factors *in vitro*.

## Materials and Methods

### Patients and Samples

Prior to their surgery, all patients enrolled in this study provided written consent for sample collection, storage, and analysis under a protocol approved by the Institutional Ethics Review Board of the First Affiliated Hospital of Dalian Medical University (Dalian, China). AD specimens were collected from 5 patients who underwent acute Stanford A dissection repair operations. For the control group, normal aortic samples were obtained from thoracic aorta tissue samples from 5 patients, discarded due to perforation of the thoracic aortic wall during coronary artery bypass grafting in the same period. The two groups were matched according to age, sex, and race matched controls.

### Animal Model

ApoE−/− mice and SPF mouse diet were purchased from Beijing Vital River Lab Animal Technology Co., Ltd. (Beijing, China). The experimental mice were fed a conventional and standard SPF mouse diet (contained 5.3% fat, 23.6% protein, and 2.9% crude fiber) and were provided with clean drinking water and housed under a 12 h light-dark cycle at 24°C. At 8 weeks age, 40 male mice were randomly divided into the following four groups (*n* = 10/group): Control, Control + M5Ab, Ang II, and Ang II + M5Ab group. ApoE−/− mice were anesthetized with an intraperitoneal injection of 50 mg/kg pentobarbital sodium and then subcutaneously implanted Alzet osmotic minipumps (Alzet model 2004; Durect Corporation, Cupertino, CA, United States) through an incision in the neck of the mouse. Minipumps were filled with either saline (control and control + M5Ab groups) or Ang II (Ang II and Ang II + M5Ab groups; Ang II human, HY-13948, MedChem Express, Princeton, NJ, United States; 1,000 ng/kg/min) solution for up to 4 weeks (Liu et al., 2015; He et al., 2019). M5Ab (CVDVPNGRGDSLAYGLRS) was purchased from HuaAn Biotechnology (Hangzhou, China), 400 μg of which was dissolved in 200 μL of phosphate-buffered saline (PBS). M5Ab was administered to mice tails intravenously (control + M5Ab and Ang II + M5Ab groups; 400 μg each injection, two injections per week for the final 2 weeks after implantation of Alzet osmotic minipumps) (Cui et al., 2019).

All mice were euthanized after a 28-day infusion, and whole aortas, including thoracic and abdominal aortas, were collected for morphological and histological analysis of the AAA. All animal experiments were performed in accordance with the Guide for the Care and Use of Laboratory Animals. The study was approved by the ethical committee of Dalian Medical University.

### Echocardiography

Mice were subjected to abdominal aorta ultrasound using a Vevo 1100 high-frequency imaging system (Visual Sonics System, Toronto, Canada) as previously described (Philips et al., 2018). The ultrasonic probe was fixed on the top of the operating table and moved to an appropriate position to avoid excessive pressure on the abdominal cavity and ensure a clear image in the abdominal cavity. The size and transverse diameter of the abdominal aorta were explored below the renal artery and above the branch of the iliac artery. The largest transverse diameter was measured three times. Repeated measurements can compress aneurysms, which leads to rupture. The mice were fasted for 12 h before ultrasound examination. The operation process lasted for 5–10 min.

### Measurement of Blood Pressure

Systolic blood pressure (SBP) was measured five times per mouse using the indirect tail-cuff method with a non-invasive sphygmomanometer (Softron BP98A, Softron Co., Ltd., Tokyo, Japan).

### ELISA

Blood samples were collected via carotid-artery and serum was prepared by centrifugation at 3,000 rpm for 15 min. Following the manufacturer’s instructions, serum concentrations of OPN-N were measured using the Mouse Osteopontin N-Half ELISA Kit (Immuno-Biological Laboratories Co., Ltd., United States).

### Histological Analysis

Whole aortas were removed, fixed in 4% paraformaldehyde for 24 h, embedded in paraffin, and 4 μm cross-sections were prepared for histological and morphological analysis. Sections of human aortic tissue were also used. Aortic sections were stained with hematoxylin and eosin (H&E) for morphological assessment in accordance with the standard procedure (Cui et al., 2019; Schack et al., 2020). The elastic fiber integrity of the abdominal aorta was visualized using Masson’s trichrome and EVG staining kit (Servicebio Biotechnology Co., Ltd., Wuhan, China) according to the manufacturer’s instructions.

### Immunohistochemistry

Paraformaldehyde-fixed paraffin-embedded sections were incubated with primary antibodies overnight at 4°C. The primary antibodies used were against OPN-N (rabbit anti-OPN-N antibody, ab181440, 1:200; Abcam, United Kingdom), NLRP3 (rabbit anti-NLRP3 antibody, 1:200; Boster, Wuhan, China), ASC (rabbit anti-ASC antibody, 10500-1-AP, 1:200; Proteintech, Wuhan, China), IL-18 (rabbit anti- IL-18 antibody, 1:200; Proteintech), IL-1β (rabbit anti-IL-1β antibody, 1:200; Arigo, Hamburg, Germany), Caspase-1 (rabbit anti- Caspase-1 antibody, 22915-1-AP, 1:200; Proteintech), MMP2 (rabbit anti-MMP2 antibody, 1:200; Proteintech), MMP9 (rabbit anti-MMP9 antibody, 1:200; Proteintech), α-smooth muscle actin (SMA) (rabbit anti-α-SMA, 1:200; Proteintech) and α9β1 (rabbit anti-α9β1, ab27947, 1:200; Abcam). As negative controls, species and isotype-matched IgG was applied in place of the primary antibody. Immunohistochemical analysis was performed using the SP-9001 SPlink Detection kits (OriGene Technologies, Inc.), according to the manufacturers’ instructions. All sections were examined using a Nikon E100 microscope. Three sections were randomly selected from each group.

### Immunofluorescence Staining (Paraffin-Slides)

Abdominal aortas were frozen and embedded in optimal cutting temperature compound. Sections of human aortic tissue were also used. Tissue sections (4 μm) were incubated with primary antibodies against α-SMA (1:200, Proteintech), OPN-N (1:200, Abcam), and α9β1 (1:200, Abcam) in a humidified chamber overnight at 4°C. Next, the sections were washed three times with PBS (PH 7.4) and incubated with the appropriate secondary antibodies at room temperature for 50 min in dark conditions. Nuclei were stained with 4’,6-diamidino-2-phenylindole (DAPI). Immunofluorescence was visualized using Nikon Eclipse C1 light microscope.

### Cell Culture

VSMCs derived from embryonic rat thoracic aorta (A7r5) were purchased from the National Collection of Authenticated Cell Cultures (Shanghai, China). The cells were maintained in Dulbecco’s modified Eagle’s medium (C11995500BT, Gibco, Thermo Fisher Scientific, Grand Island, NY, United States) containing 10% fetal bovine serum, 100 U/mL penicillin, and 100 μg/mL streptomycin. The VSMCs were maintained at 37°C in an atmosphere of 95% air and 5% CO_2_. Media were changed every other day, and cells were passaged at least twice a week (Wang F. et al., 2020). Passaging was accomplished by the addition of a trypsin/EDTA solution (HyClone, SH30042.01) in PBS and collection of cells by centrifugation (1,000 rpm, 5 min). To prepare OPN-N, 10 μg of recombinant human (rh) osteopontin OPN (R&D Systems,1433-OP-005/CF, Minneapolis, MN, United States) was added with 1 U thrombin (sigma-Aldrich) and incubated at 37°C for 1 h, stored at -80°C, and thawed to room temperature when used (Cui et al., 2019). Cells were grown to 80–90% confluence and treated with OPN-N (0–0.6 μg/mL) for up to 12 (for RT-PCR) or 24 h (for Western blotting). SN50 is a cell permeable inhibitor of NF-κB translocation. SN50 was purchased from MedChem Express (HY-P0151, Princeton, NJ, United States).

### Immunofluorescence Staining (Cell Climbing Slides)

VSMCs were grown on glass coverslips in 6-well plates with a density of 1 × 10^6^/well and treated with OPN-N for 24 h. Cells were washed with PBS and fixed in 4% paraformaldehyde, and permeabilized using permeabilize working solution. Then, VSMCs were blocked with 3% BSA and incubated with primary antibodies against α-SMA (rabbit anti-α-SMA, 1:200; Proteintech), NLRP3 (rabbit anti-NLRP3 antibody, 1:100; Boster), IL-1β (rabbit anti-IL-1β antibody, 1:200; Arigo), IL-18 (rabbit anti- IL-18 antibody, 1:200; Proteintech), and Caspase-1 (rabbit anti- Caspase-1 antibody, 1:200; Proteintech) over night at 4°C (diluted with PBS appropriately). After washing with PBS, the cells were incubated with secondary antibody at room temperature for 50 min, then with DAPI solution in a dark for an additional 10 min and washed. Slides were mount with anti-fade mounting medium, then detected and collected images using Fluorescent Microscopy (Nikon Eclipse C1).

### RNA Isolation and Real-Time PCR

Total RNA was isolated from aortic tissue or VSMCs and complementary DNA (cDNA) using the TransScript One-Step gDNA Removal and cDNA Synthesis SuperMix kit (Transgen, Beijing, China) according to the manufacturer’s instructions. Gene expression was analyzed quantitatively using qPCR using the TransStart Top Green qPCR SuperMix kit (Transgen). β-Actin cDNA was amplified and quantitated in each cDNA preparation to normalize the relative expression of the target genes. Primer sequences are listed in Table 1.

**TABLE 1:** Primer oligonucleotide sequences.

Gene	Primers
Mouse TNF-α	F:5′-ACGGCATGGATCTCAAAGAC-3′
	R:5′-GTGGGTGAGGAGCACGTAGT-3′
Mouse IL-6	F:5′-CTGCAAGAGACTTCCATCCAGTT-3′
	R:5′-GAAGTAGGGAAGGCCGTGG-3′
Mouse MCP-1	F:5′-ACCTGCTGCTACTCATTCAC-3′
	R:5′-CATTCAAAGGTGCTGAAGAC-3′
Mouse β-actin	F:5′-AGCTTACTGCTCTGGCTCCTAGC-3′
	R:5′-ACTCATCGTACTCCTGCTTGCT-3′
Rat NLRP3	F:5′-CGGTGACCTTGTGTGTGCTT-3′
	R:5′-TCATGTCCTGAGCCATGGAAG-3′
Rat IL-1β	F:5′-CCTATGTCTTGCCCGTGGAG-3′
	R:5′-CACACACTAGCAGGTCGTCA-3′
Rat Caspase-1	F:5′-GAACAAAGAAGGTGGCGCAT-3′
	R:5′-AGACGTGTACGAGTGGGTGT-3′
Rat IL-18	F:5′-ACCACTTTGGCAGACTTCACT-3′
	R:5′-ACACAGGCGGGTTTCTTTTG-3′
Rat GAPDH	F:5′- CTGGAGAAACCTGCCAAGTATG-3′
	R:5′-GGTGGAAGAATGGGAGTTGCT-3′

*Abbreviations: TNF-α, tumor necrosis factor-α; IL-6, interleukin- 6; IL-1β, interleukin- 1β.*

### Western Blotting Analysis

Total protein was extracted from the aortas of ApoE^–/–^ mice, human aortic tissue or VSMCs. Western blotting was performed according to standard protocols. Total proteins were extracted using RIPA assay and the concentration measured using the BCA protein assay kit (Servicebio Biotechnology Co., Ltd., Wuhan, China). Protein samples were separated using SDS-PAGE and transferred into PVDF membranes. After quick washing with TBST, the transferred membrane was blocked in 5% non-fat milk for 0.5 h and incubated overnight at 4°C with the primary antibodies: rabbit anti-β-Actin (1:1,000, Proteintech), rabbit anti-OPN-N (1:1,000, Abcam), rabbit anti-NLRP3 (1:1,000, Boster), rabbit anti-IL-18 (1:1,000, Proteintech), rabbit anti-IL-1β (1:500, Arigo), rabbit anti-Caspase-1 (1:1000, Proteintech), rabbit anti-Gasdermin D (GSDMD, 1:1000, Affinity Biosciences, OH, United States), rabbit anti-MMP2 (1:1,000, Proteintech), and rabbit anti-MMP9 (1:1,000, Proteintech). Washing three times with TBST for 5 min each time and incubation with specific conjugated secondary antibodies (1:5000 dilution in TBST) for 30 min at room temperature. Repeat washing the film three times for 5 min each with TBST, the immunoreactive bands were visualized using ECL reagents (Millipore Corp., MA, United States). Protein levels are expressed as protein/β-actin ratios to minimize loading differences. The relative signal intensity was quantified using Image J software.

### Statistical Analysis

Statistical analyses involved use of Graphpad Prism 5.0. All data are presented as mean ± SEM. Statistical differences between two groups were analyzed by the unpaired Student’s *t* test and differences between multiple groups of data were analyzed by One-way and Two-way ANOVA. *P* < 0.05 was considered statistically significant.

## Results

### OPN-N Expression and Histological Changes in AD Tissues and Normal Aortas

**FIGURE 1:**
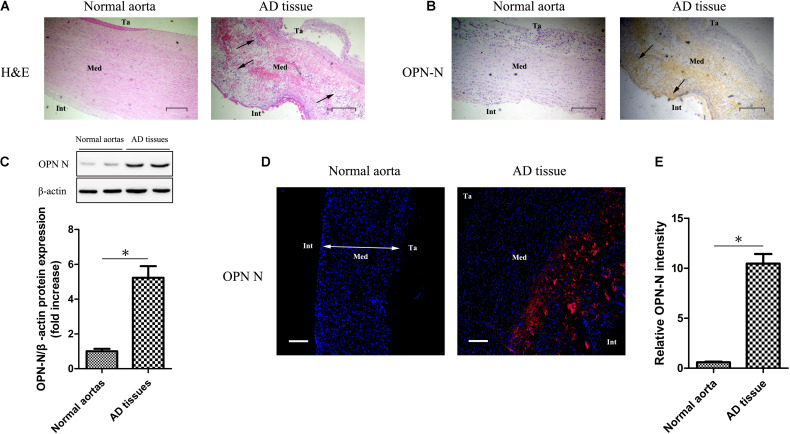
OPN-N expression and histological changes in AD tissues and normal aortas. **(A)** H&E staining of AD tissue and normal aorta (Scar bar 400 μm). **(B)** Immunohistochemistry staining for OPN-N in AD tissue and normal aorta (Scar bar 200 μm). **(C)** Western blotting for OPN-N in AD tissues and normal aortas, bar graph showing quantification of OPN-N protein expression. **(D,E)** Representative immunofluorescence staining for OPN-N in AD tissue and normal aorta **(D)** and quantification of OPN-N intensity **(E)** (Scar bar 200 μm; DAPI staining of nuclei: blue; OPN-N staining: red). β-actin was used as an internal control. Data are expressed as means ± SEM; *n* = 5 per group. ^∗^*P* < 0.05, unpaired Student’s *t* test. Abbreviations: AD, aortic dissection; H&E, hematoxylin and eosin; OPN-N, osteopontin N-terminal fragment; Ta, tunica adventitia; Int, intima; and Med, media.

H&E staining (Figure 1A) showed significant inflammatory cell infiltration in the medial layer (black arrows), deletion of smooth muscle cells, and decrease in nuclei and vacuolization in AD tissue with that in normal aorta. Using immunohistochemistry (Figure 1B), western blotting (Figure 1C), and immunofluorescence (Figures 1D,E), we observed that OPN-N expression was significantly higher in the AD tissues than in the normal aortas. Normal aorta showed a very weak OPN-N staining.

### Administration of M5Ab Attenuates the Expression of OPN-N in ApoE−/− Mice With AAA

**FIGURE 2:**
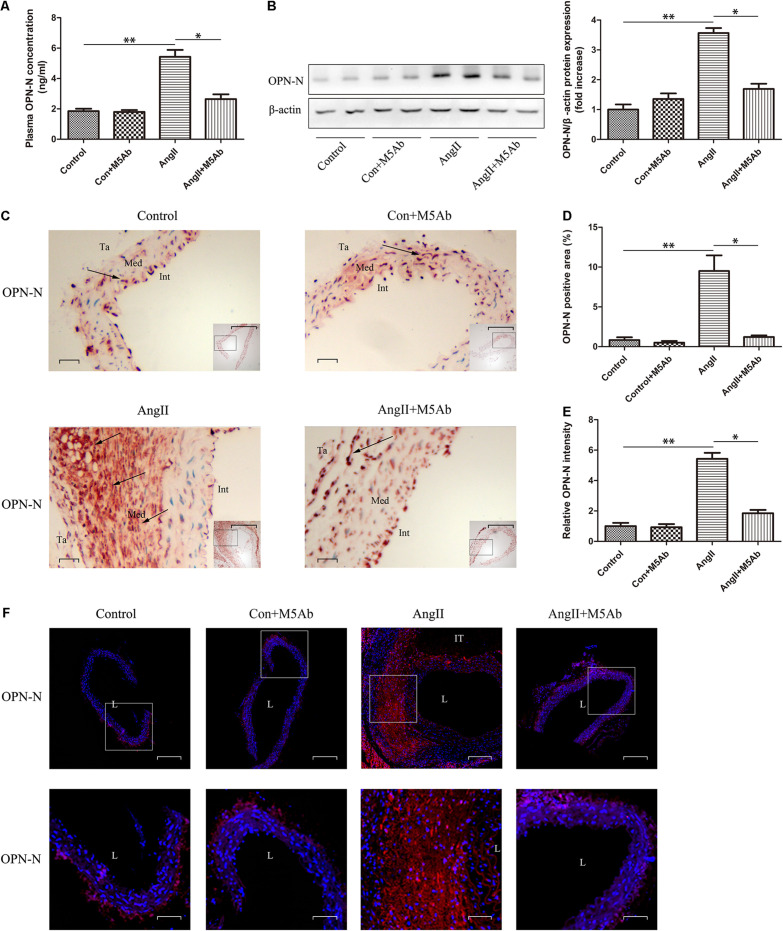
Administration of M5Ab attenuates the expression of OPN-N in ApoE−/− mice with AAA. **(A)** ELISA analyses of the levels of serum OPN-N in mice under different treatments. **(B)** Western blotting for OPN-N in mice under different treatments (left), bar graph showing quantification of OPN-N protein expression (right). β-actin was used as an internal control. **(C,D)** Representative images of aortic immunohistochemistry staining to detect OPN-N expression in mice (black arrows), quantification of OPN-N area. Scar bar 200 μm, boxed areas; Scar bar 50 μm, main images. **(E,F)** OPN-N immunofluorescence staining of aortic tissues in mice and quantification of OPN-N intensity (Scar bar 50 μm), boxed areas in F are shown at higher magnification below (Scar bar 20 μm). Data are expressed as means ± SEM; *n* = 3 per group. ^∗^*P* < 0.05 vs the Ang II + M5Ab group; ^∗∗^*P* < 0.05 vs the control and control + M5Ab groups. Abbreviations: Ang II, angiotensin II; OPN-N, osteopontin N-terminal fragment. DAPI staining of nuclei: blue; OPN-N staining: red. Ta, indicates tunica adventitia; Int, intima; Med, media; L, lumen; and IT, intraluminal thrombus.

To investigate the role of OPN-N in the development of Ang II-induced AAA in mice, we used ELISA (Figure 2A), western blotting (Figure 2B), immunohistochemistry (Figures 2C,D), and immunofluorescence staining (Figures 2E,F) to determine the expression of the OPN-N. We observed that Ang II infusion, over 28 days, upregulated OPN-N levels in ApoE−/− mice when compared with the control group. Meanwhile, M5Ab inhibited the expression of OPN-N in the Ang II + M5Ab group, in comparison with the Ang II group, indicating that administration with M5Ab attenuated the effect of OPN-N in Ang II-induced AAA. A negative control used rabbit IgG and yielded no signal in AAA lesions (Supplementary Figure 2).

### Characteristics of AngII-Induced AAA Model Ultrasonography and Baseline

**FIGURE 3:**
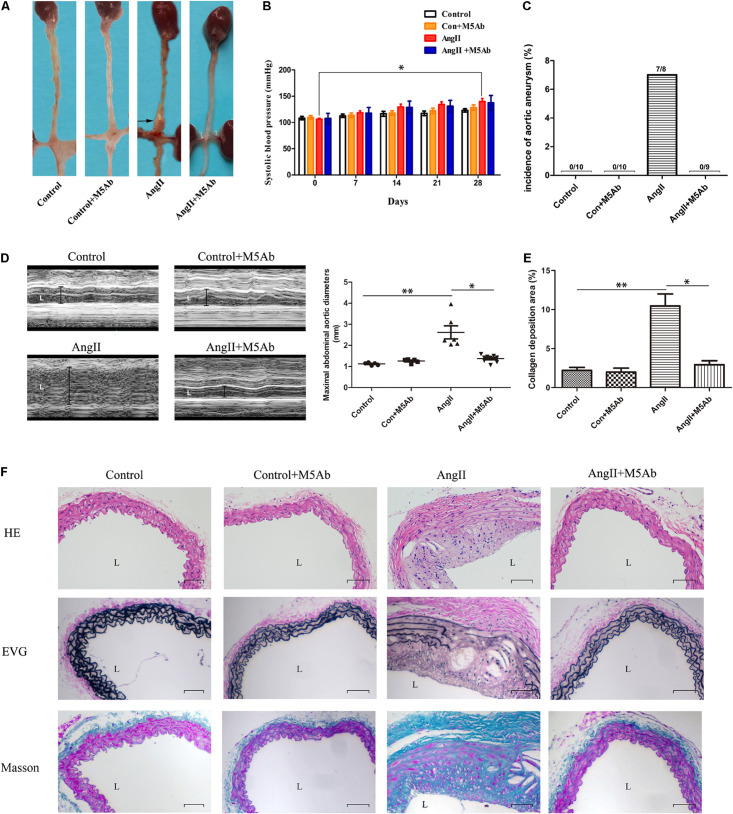
Characteristics of AngII-induced AAA model ultrasonography and baseline. **(A)** Representative the macroscopic features of abdominal aorta in the four groups. **(B)** The SBP was measured by tail-cuff weekly in the four groups. Baseline blood pressure was not different. Compared with that on the 0th day, the SBP in the AngII group increased on the 28th day, which was statistically significant (123 ± 3.25 mmHg vs 139 ± 5.83 mmHg, *P < 0.05, two-way ANOVA), while the SBP among the four groups was not statistically significant in the weekly comparison (P > 0.05, two-way ANOVA). *n* = 5 per group. **(C)** Percentage of mice in which aortic aneurysm was successfully achieved in the four groups. **(D)** Representative the ultrasonography images (left) and the data of maximal abdominal aortic diameters (right) in the four groups at the end of 28 days. **(E,F)** The histological analysis (H&E, EVG, and Masson’s trichrome staining) of abdominal aorta of mice under different treatments for each condition (Scar bar 100 μm). Statistical analysis of collagen deposition. *P < 0.05 vs the Ang II + M5Ab group; **P < 0.05 vs the control and control + M5Ab groups. Abbreviations: H&E, hematoxylin and eosin; and L, lumen. Data are expressed as means ± SEM; *n* = 3 per group.

AAA was induced after 28 days of Ang II infusion (Figure 3A). Tail-cuff SBP was measured, and there was no statistical difference in SBP among the four groups each week. Except for the comparison between the 0th day and the 28th day in the Ang II group, there was no significant difference in intra-group comparison between weeks (Figure 3B). No aortic aneurysm had occurred in either control or control + M5Ab groups. In the Ang II group, seven mice had developed aneurysm in the suprarenal region of the aorta (two had died due AAA rupture) and one developed aortic arch aneurysm. One mouse in the Ang II + M5Ab group had died accidentally during surgery after implanted with minipumps, and none of the mice in this group achieved the AAA standard (Figure 3C). M-mode ultrasound showed no differences in the size of the abdominal aorta between the control (1.12 ± 0.06 mm) and control + M5Ab (1.26 ± 0.10 mm) groups. The abdominal aorta of the Ang II-treated mice was larger than that of the control (2.62 ± 0.76 mm versus 1.12 ± 0.06 mm; *P* < 0.05, Figure 3D), however, M5Ab treatment mitigated this increase in the Ang II + M5Ab mice (1.37 ± 0.16 mm) group (P < 0.05, Figure 3D).

### M5Ab Reduces Inflammatory Cell Infiltration, Degradation of Elastic Fibers, and Collagen Deposition by Blocking the Expression of OPN-N

Histological changes in the abdominal aorta are shown in Figures 3E,F. H&E staining showed significant histopathological changes in the Ang II group compared with the control, such as arterial wall injury and inflammatory cell infiltration. Moreover, this change was significantly suppressed in the Ang II + M5Ab group. Meanwhile, EVG staining showed extensive degradation of the elastic fibers or focal breakage of elastin in the aneurysmal aortic tissues of the Ang II group. The degradation of elastic fibers is a hallmark of AAA (Latz et al., 2013; Kono et al., 2014). Mice treated with M5Ab had slightly better structural elastin fibers than those in the Ang II group. Additionally, the sections were stained with Masson’s trichome to identify collagen fibers (blue), muscle fibers (purple red), cellulose and red blood cells (red). Masson’s trichrome staining showed significantly more collagen deposition in the Ang II group than in the control group, suggesting that Ang II treatment induced abdominal aorta collagen deposition, which was markedly reduced by M5Ab treatment (Schack et al., 2020). These histological changes indicated that OPN-N has a positive association with the progression of AAA.

### M5Ab Inhibits Ang II-Induced Expression of Pyroptosis-Related Inflammatory Factors in Mice by Blocking the Expression of OPN-N

**FIGURE 4:**
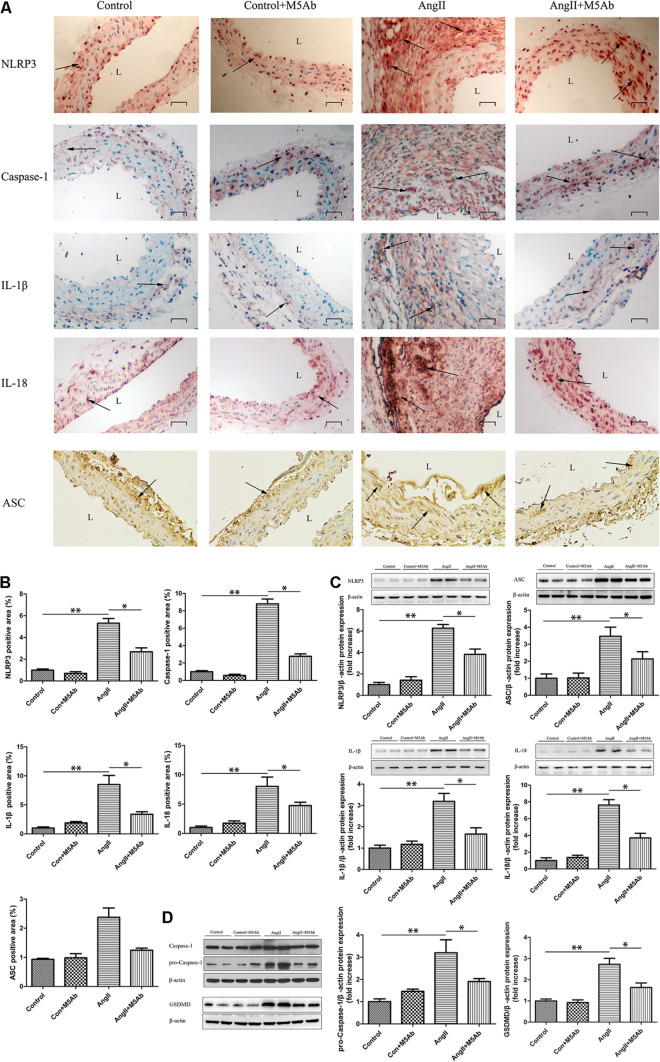
M5Ab inhibits Ang II-induced expression of pyroptosis-related inflammatory factors in mice by blocking the expression of OPN-N. **(A,B)** Immunohistochemistry staining of NLRP3, Caspase-1, ASC, IL-1β, and IL-18 in mice under different treatments, quantification of these pyroptosis-related inflammatory factors area (below). Scar bar 25 μm. **(C,D)** Western blotting analyses of the protein levels of NLRP3, ASC, IL-1β, IL-18, pro-Caspase-1, and GSDMD in mice, and quantification of these pyroptosis-related inflammatory proteins expression. β-actin was used as an internal control. Data are expressed as means ± SEM; *n* = 3 per group. ^∗^*P* < 0.05 vs the Ang II + M5Ab group; ^∗∗^*P* < 0.05 vs the control and control + M5Ab groups. Abbreviations: Ang II, angiotensin II; IL, interleukin; NLRP3, NOD-, LRR-, and pyrin domain-containing protein 3; and L, lumen.

Since pyroptosis and inflammation plays an important role in aortic aneurysm, we next investigated the association between pyroptosis-related inflammatory factors and OPN-N in Ang II-induced AAA mouse model. Immunohistochemistry staining and western blotting were conducted on pyroptosis-related factors (including NLRP3, pro-Caspase-1/Caspase-1, ASC, GSDMD) and inflammatory factors like IL-1β and IL-18. Immunohistochemistry staining showed that, in the Ang II + M5Ab group, the expression of NLRP3, Caspase-1, ASC, IL-1β, and IL-18 was markedly lower than that in the Ang II group (Figures 4A,B). Similarly, western blotting showed that the protein levels of pyroptosis-related protein (NLRP3, ASC, GSDMD, and pro-Caspase-1) and the inflammatory factors (IL-1β and IL-18) were lower in the Ang II + M5Ab group than in the Ang II group (Figures 4C,D). These results showed that OPN-N inhibition downregulated the expression of pyroptosis-related inflammatory factors.

### M5Ab Attenuates Proinflammatory Cytokine Gene Expression and Inflammatory Cell Infiltration by Blocking the Expression of OPN-N

**FIGURE 5:**
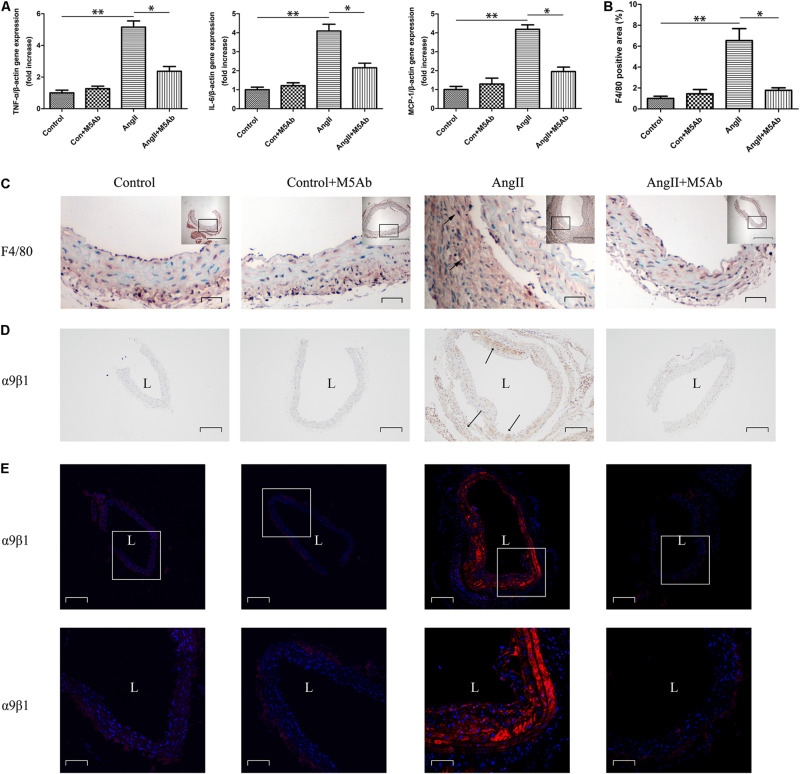
M5Ab attenuates proinflammatory cytokine gene expression and inflammatory cell infiltration by blocking the expression of OPN-N. **(A)** qPCR analyses of the mRNA expression levels of TNF-α, IL-6, and MCP-1 in mice under different treatments. **(B,C)** Immunohistochemistry staining of F4/80 macrophages in mice under different treatments, quantification of these F4/80 area. Scar bars: 200 μm (boxed areas), 25 μm (main images). **(D)** Immunohistochemistry staining and **(E)** Immunofluorescence staining of α9β1 in mice under different treatments. Scar bars: 200 μm (above), 50 μm (below). DAPI staining of nuclei:blue; α9β1 staining:red. Data are expressed as means ± SEM; *n* = 3 per group. ^∗^*P* < 0.05 vs the Ang II + M5Ab group; ^∗∗^*P* < 0.05 vs the control and control + M5Ab groups. Abbreviations: Ang II, angiotensin II; IL, interleukin; MCP, monocyte chemoattractant protein; TNF, tumor necrosis factor; L, lumen.

Administration of Ang II increased the gene expression levels of proinflammatory cytokines, such as TNF-α, IL-6, and monocyte chemoattractant protein (MCP)-1. However, this increase was attenuated by M5Ab in Ang II + M5Ab mice group (Figure 5A). Furthermore, immunohistochemistry staining of F4/80 macrophages showed that Ang II enhanced macrophage recruitment in adventitia, whereas M5Ab treatment attenuated inflammatory cell infiltration by blocking the expression of OPN-N in Ang II + M5Ab mice group (Figures 5B,C). As mentioned above, OPN-N exposes an additional cryptic integrin binging motif that binds to integrins thereby enhancing proinflammatory effect. Expression of α9β1 integrin exhibited a dramatic increase in Ang II group. Immunohistochemistry (Figure 5D) and immunofluorescence (Figure 5E) staining showed that, in the Ang II + M5Ab group, the expression of α9β1 was markedly lower than that in the Ang II group.

### M5Ab Inhibits MMP2 and MMP9 Expression in Mice by Blocking the Expression of OPN-N

**FIGURE 6:**
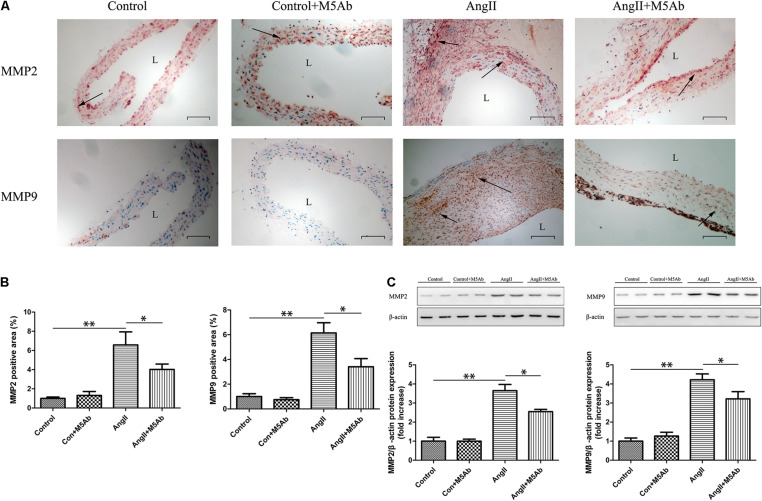
M5Ab inhibits MMP2 and MMP9 expression in mice by blocking the expression of OPN-N. **(A,B)** Immunohistochemistry staining of MMP2 and MMP9 in mice under different treatments **(A)**, quantification of MMP2 and MMP9 area **(B)**. Scar bar 100 μm. **(C)** Western blotting analyses the protein levels of MMP2 and MMP9 in mice, and quantification of MMP2 and MMP9 protein expression. β-actin was used as an internal control. Data are expressed as means ± SEM; *n* = 3 per group. ^∗^*P* < 0.05 vs the Ang II + M5Ab group; ^∗∗^*P* < 0.05 vs the control and control + M5Ab groups. Abbreviations: Ang II, angiotensin II; MMP, matrix metalloproteinase; and L, lumen.

Immunohistochemistry showed that, in the Ang II + M5Ab group, the expression of MMP2 and MMP9 was markedly lower than that in the Ang II group (Figures 6A,B). Western blotting showed that the expression of MMP2 and MMP9 was lower in the Ang II + M5Ab than in the Ang II group (Figure 6C). These results indicated that OPN-N promoted the formation of AAA by increasing MMP2 and MMP9 levels.

### M5Ab Decreases the Downregulation of α-SMA in the AAA Group Mice

**FIGURE 7:**
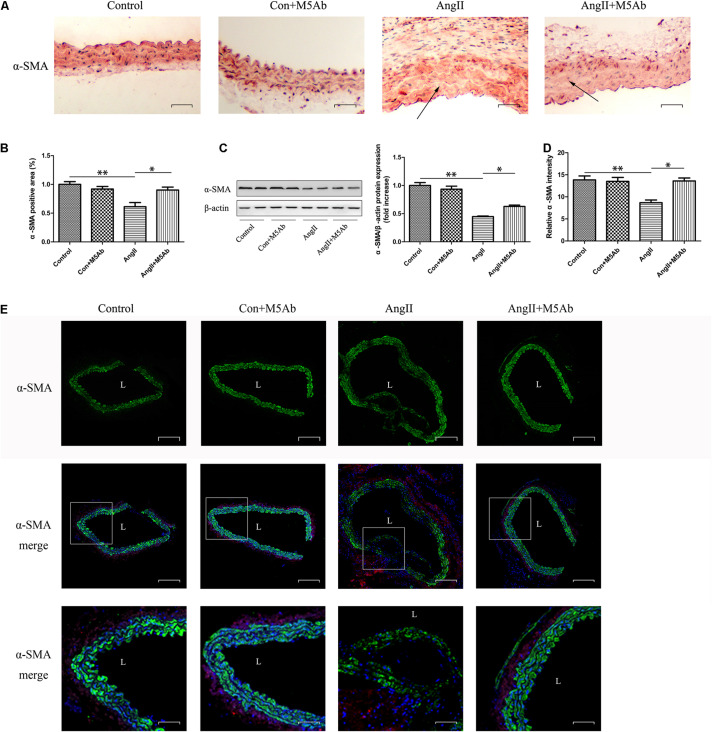
M5Ab decreases the downregulation of α-SMA in the AAA group mice. **(A,B)** Immunohistochemistry staining of α-SMA in mice under different treatments **(A)**, quantification of α-SMA area **(B)**. Scar bar 25 μm. **(C)** Western blotting analyses of the protein levels of α-SMA in mice, and quantification of α-SMA protein expression. **(D,E)** Representative α-SMA immunofluorescence staining of aortic tissues and quantification of α-SMA intensity (Scar bar 50 μm), boxed areas in panel **(E)** are shown at higher magnification below (Scar bar 20 μm). Data are expressed as means ± SEM; *n* = 3 per group. ^∗^*P* < 0.05 vs the Ang II + M5Ab group; ^∗∗^*P* < 0.05 vs the control and control + M5Ab groups. Abbreviations: Ang II, angiotensin II; SMA, smooth muscle actin; and L, lumen.

Representative immunohistochemistry and immunofluorescence images of α-SMA in mice are shown in Figures 7A,B,D,E, respectively. In the control group, the smooth muscle thickness was uniform with a regular shape and cells were intact with no obvious pathological changes. The smooth muscle arrangement in the Ang II group was disordered, the density was decreased, and some cells were dissolved. The expression of α-SMA in the Ang II + M5Ab group was markedly higher than that in the Ang II group, while the protein levels of α-SMA, determined using western blotting, were higher in the Ang II + M5Ab group than in the Ang II group (Figure 7C). These results indicated that OPN-N promoted the formation of AAA by decreasing α-SMA levels and M5Ab decreased the downregulation of α-SMA in mice by blocking the expression of OPN-N.

### *In vitro* Studies

**FIGURE 8:**
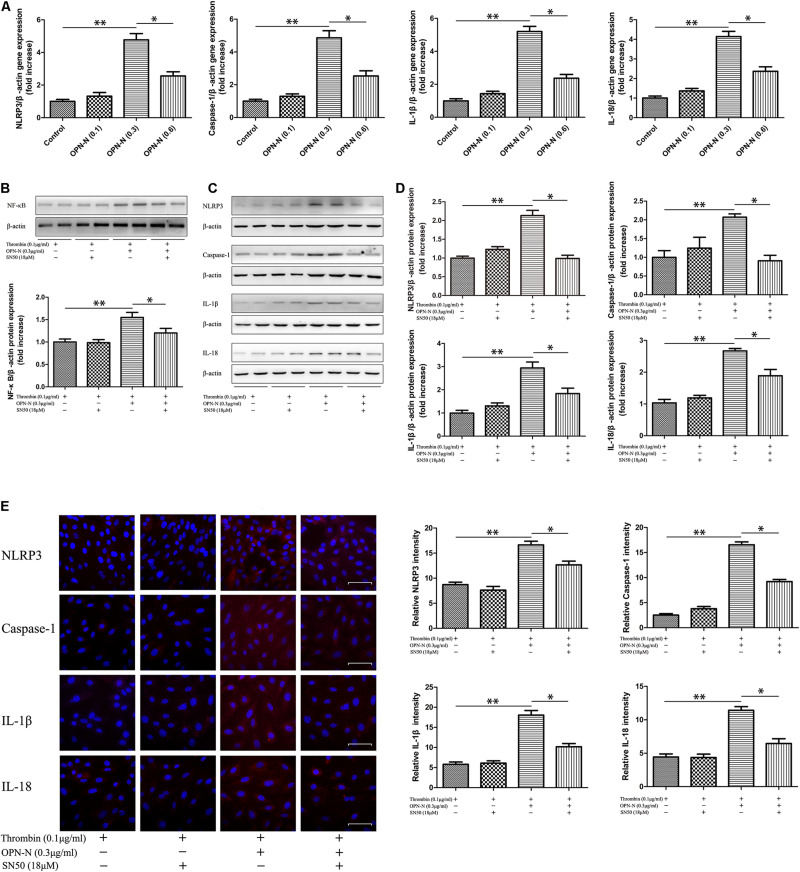
OPN-N had a causal link of pyroptosis and inflammation through NF-KB mediated signaling pathways in VSMCs. **(A)** qPCR analyses of the mRNA expression level of NLRP3, Caspase-1, IL-1β, and IL-18 at different OPN-N concentrations. **(B)** Western blotting analyses of the protein levels of NF-κB in VSMCs, and quantification of NF-κB protein expression. **(C,D)** Western blotting analyses of the protein levels of NLRP3, Caspase-1, IL-1β, and IL-18 in VSMCs **(C)**, and quantification of these pyroptosis-related inflammatory factors protein expression **(D)**. β-actin was used as an internal control. **(E)** Immunofluorescence staining of NLRP3, Caspase-1, IL-1β, and IL-18 in mice under different treatments (left), quantification of these pyroptosis-related inflammatory factors area (right). Scar bar 50 μm. Data are expressed as means ± SEM; *n* = 3 per group. ^∗^*P* < 0.05 vs the Thrombin + OPN-N + SN50 group; ^∗∗^*P* < 0.05 vs the Thrombin and Thrombin + SN50 groups. Abbreviations: IL, interleukin; NF, nuclear factor; NLRP3, NOD-, LRR-, and pyrin domain-containing protein 3; OPN-N, osteopontin N-terminal fragment.

To further investigate the link between OPN-N and pyroptosis-related inflammatory factors (NLRP3, Caspase-1, IL-1β, and IL-18), cultured VSMCs were incubated with OPN-N (0–0.6 μg/mL) for up to 12 h. When the OPN-N concentration was 0.3 μg/mL, gene expressions of pyroptosis-related inflammatory factors were significantly increased in a non-dose-dependent manner (Figure 8A). Following treatment with 0.3 μg/mL OPN-N for 24 h, the protein levels of nuclear factor (NF) -κB were higher in the OPN-N group than in the Thrombin group (0.1 μg/mL); reciprocally, this increase was suppressed by preincubation with SN50 (18 μM) for 1 h (Figure 8B). We next determined the effect of OPN-N on the NF-κB signaling and pyroptosis-related inflammatory factors expression. Western blotting (Figures 8C,D) and immunofluorescence staining (Figure 8E) showed that the expression of pyroptosis-related inflammatory factors was lower in the OPN-N + SN50 group than in the OPN-N group. These results demonstrated that the NF-κB is involved in OPN-N induced pyroptosis in VSMCs.

## Discussion

**FIGURE 9:**
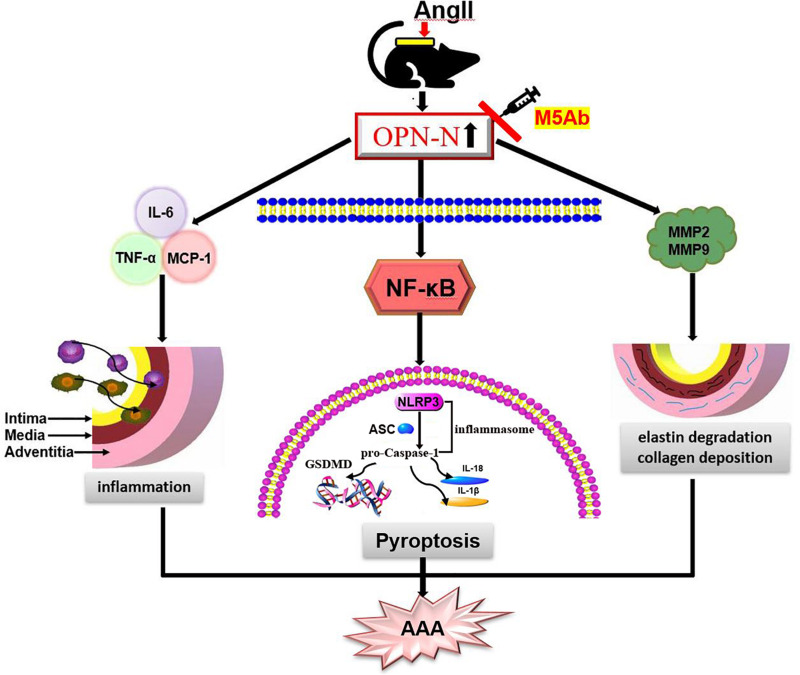
Schematic diagram of the function of OPN-N in the AngII-induced AAA. OPN-N as a pivotal pro-inflammatory cytokine induces pyroptosis and inflammation through the activation of its NF-κB pathway aggravates AAA formation. OPN-N influences aneurysm formation in multiple ways, such as increasing the expression of TNF-α, IL-6, and MCP-1 and enhancing the production of MMP2 and MMP9, which further activates the elastin degrade and increases compensatory collagen deposition. Abbreviations: AAA, abdominal aortic aneurysm; OPN-N, osteopontin N-terminal fragment.

The present study showed that the levels of OPN-N are significantly increased in Ang II-induced AAA animal model and the aortic tissue of AD patients. Herein, by blocking the function of OPN-N using M5Ab, we investigated the expression down regulation of pyroptosis-related inflammatory factors including NLRP3, pro-Caspase-1, ASC, GSDMD, IL-1β, and IL-18 in Ang II treatment mouse model. Furthermore, treatment of VSMCs with OPN-N markedly stimulated pyroptosis-related inflammatory proteins expression, reciprocally, the effects were markedly attenuated by the inhibition of NF-κB. These findings indicated that OPN-N had a causal link with pyroptosis through NF-κB mediated signaling pathways in VSMCs (Figure 9).

Several studies have reported the role of OPN in inflammation and fibrosis regulation in human aortic aneurysm disease and animal models (Bruemmer et al., 2003; Golledge et al., 2007; Zheng et al., 2012; Fan et al., 2019), however, the structural subtype or domain of OPN that participates in or promotes the progress of AAA formation remains to be determined. OPN-N has stronger pro-inflammatory effect than the intact structure of OPN, and influences the progression of liver fibrosis in mouse models of liver fibrosis and liver fibrosis patients (Cui et al., 2019). A functional serine-valine-valine-tyrosine-glutamate-leucine-arginine (SVVYGLR) motif of the OPN-N fragment attributes to the OPN-N proinflammatory property (Sharony et al., 2010) and enhancements of the myocardial fibrosis in the rat model of myocardial fibrosis (Uchinaka et al., 2015). In our study, we compared aortic tissue level of OPN-N expressions between AD patients and healthy controls and found that OPN-N expression was significantly increased in the AD patients compared to that in healthy controls, and that normal aorta showed very weak OPN-N staining. This result suggests that OPN-N may be involved in the formation of aortic aneurysm disease.

M5Ab, a specific anti-OPN-N antibody used to investigation the function of OPN-N in certain diseases, has been shown to inhibit SLAYGLR of OPN-N in rheumatoid arthritis mouse model (Yamamoto et al., 2003). Therefore, we investigated the role of OPN-N in AAA by establishing an Ang II-induced AAA mouse model. Histologically, AAA is characterized by the loss of VSMCs, inflammatory cell infiltration, degradation of elastic fibers, and compensatory collagen deposition as previously reported (Latz et al., 2013; Kono et al., 2014). The ultrasound images showed a significant expansion of the abdominal aorta in the Ang II group compared with the control group, which indicated that AAA was established in mice using Ang II infusion. We also observed that the expression of OPN-N was higher in the Ang II than in the control group. Following M5Ab treatment, the incidence of aortic aneurysms, the maximal abdominal aortic diameter, and the histopathological damage changes in the Ang II + M5Ab group were lower than those in the Ang II group. These results indicated that OPN-N was positively correlated with the Ang II-induced AAA mouse model.

Pyroptosis is a highly pro-inflammatory, programmed cell death, which plays an important role in the progression of cardiovascular diseases, such as atherosclerosis, myocardial infarction, and diabetic cardiomyopathy (O’Regan et al., 1999; Luo et al., 2014; Boggio et al., 2016; van et al., 2017; Zhou et al., 2018). Previous studies have demonstrated that the activation of NLRP3 inflammasome in macrophage and subsequent release of IL-1β are closely associated with AAA inflammatory response (Sun et al., 2015). Usui et al. (2015) found that in Ang II induced AAA model, while knockdown of NLRP3, ASC and Caspase-1 genes can reduce the incidence and severity and the initial inflammatory responses of AAA. Several studies have suggested that activated NLRP3 inflammasome and GSDMD promote the release of pro-inflammatory factors such as IL-1β and IL-18, thereby inducing cell pyroptosis (Lamkanfi and Dixit, 2012; Strowig et al., 2012; Liu et al., 2016; Takahashi, 2021). Herein, we showed that the expression of pyroptosis-related inflammatory factors such as NLRP3, pro-Caspase-1, GSDMD, ASC, IL-1β, and IL-18 were markedly higher in the Ang II group than the control group, whereas anti-OPN-N antibody attenuated this effect, thereby providing evidence supporting the causal link between OPN-N and pyroptosis-related inflammatory factors (e.g., NLRP3, pro-Caspase-1, IL-1β, and IL-18), and that OPN-N is a pro-inflammatory cytokine which stimulates pyroptosis and inflammation in Ang II-induced AAA mouse model.

Aortic aneurysm is characterized by chronic vascular inflammation and infiltration of inflammatory cells (such as macrophages, neutrophils). Inflammatory cells secrete various pro-inflammatory cytokines (MCP-1, TNF-α, IL-6, and IL-1β) to further activate VSMCs to secrete MMPs, which degrade elastin, increase compensatory collagen deposition, and damage the structure of artery walls, thereby causing lumen dilation and thrombus (Golledge et al., 2009; Rizas et al., 2009; Wang et al., 2014; Anzai et al., 2015). Administration of Ang II significantly increased inflammatory cells infiltration, as confirmed by H&E and F4/80 antibody immunohistochemical staining. This study also showed that the mRNA levels of TNF-α, IL-6, and MCP-1, using real-time PCR, was increased in the Ang II mice. Notably, the mRNA levels of TNF-α, IL-6, and MCP-1 were downregulated following M5Ab treatment. We quantitatively analyzed the protein expression of MMP2 and MMP9 using immunohistochemistry and western blotting and showed that it was higher in the Ang II group compared with the control group, which was mitigated by M5Ab treatment. This result further confirmed that OPN-N has pivotal pro-inflammatory properties that influence aneurysm formation, increase the expression of TNF-α, IL-6, and MCP-1, further activate the vascular inflammation process, and enhance MMP2 and MMP9 production.

The phenotypic transition of VSMCs plays a critical role in the formation of AAA. VSMCs homeostasis in AAA is disturbed, while VSMC phenotypic switching-mediated vascular pathology contributes to AAA formation (Davis et al., 2014). α-SMA is a cell marker derived from smooth muscle in different states of maturity (Ailawadi et al., 2009; Salmon et al., 2013). Studies have shown that the degree of OPN-N expression is positively correlated with the degree of arteriosclerosis but negatively correlated with the expression of α-SMA. In our study, we also observed that the expression of α-SMA was decreased in the Ang II group. Additionally, immunofluorescence staining showed that aortic integrity was impaired. Increasing evidence shows that VSMC loss or dysfunction aggravates inflammatory response and induces aortic aneurysm disease (Wortmann et al., 2019). To further elucidate the mechanism by which OPN-N exaggerates inflammation, we used VSMCs for *in vitro* studies. NF-κB is a family of transcription factors that regulate genes involved in inflammation and immune response, mitosis, and apoptosis (Baeuerle and Henkel, 1994; Ghosh et al., 1998; Perkins, 2000). The activation of NLRP3 inflammasome requires “priming” with Toll-like receptors agonists to initiate signaling cascades (primarily nuclear NF-κB-dependent pathways) (Bauernfeind et al., 2009). Mi et al. (2011) has demonstrated that OPN can up-regulate the expression of MMP via NF-κB signaling pathway in the thoracic aortic aneurysm and AAA. OPN activates NF-κB signaling via interactions with integrin αvβ3 (Philip and Kundu, 2003; Das et al., 2005; Qin et al., 2018). Therefore, we hypothesized that OPN-N-induced pyroptosis is caused by the activation of the NF-κB pathway, which induces the expression of the NLRP3 inflammasome. Following treatment with the NF-κB inhibitor, SN50, the expression of NLRP3, Caspase-1, IL-1β, and IL-18 was lower in the OPN-N + SN50 group than in the OPN-N group, which confirms our hypothesis. Overall, OPN-N activates the NF-κB pathway, thereby activating the NLRP3 inflammasome, which releases Caspase-1, IL-1β, and IL-18, ultimately causing pyroptosis and inflammation.

However, this study has few limitations. First, this study was performed in a mouse model of AAA which only mimics certain aspects of human disease. Second, under inflammatory conditions, OPN-N is highly expressed in macrophages, VSMCs and endothelial cells, and its mechanism involved in cellular inflammation and pyrolysis needs to be confirmed and explored. Third, further research will be needed to demonstrate whether the relevant mechanism of OPN-N in AAA is different from that of full length OPN and C-half OPN.

## Conclusion

In summary, our study provided experimental evidence that OPN-N expression in human AD tissues and Ang II-induced AAA mouse model is significantly elevated. OPN-N, as a pro-inflammatory cytokine, induces pyroptosis and inflammation through the activation of NF-κB pathway which aggravates aortic aneurysm formation. OPN-N might serve as a potential treatment target against AAA in which pyroptosis and inflammation is involved.

## Data Availability Statement

The raw data supporting the conclusions of this article will be made available by the authors, without undue reservation.

## Ethics Statement

The studies involving human participants were reviewed and approved by the Institutional Ethics Review Board of the First Affiliated Hospital of Dalian Medical University. The patients/participants provided their written informed consent to participate in this study. The animal study was reviewed and approved by the Ethical Committee of Dalian Medical University.

## Author Contributions

YJ and HL designed this study. HL, YS, and WS helped to perform the experiments. HL and YZ analyzed the data and interpreted the results of the experiments. HL and YS prepared the figures. HL drafted the manuscript. WS and YJ provided the funding for the study and helped to revise the manuscript. All authors have read and approved the final manuscript.

## Conflict of Interest

The authors declare that the research was conducted in the absence of any commercial or financial relationships that could be construed as a potential conflict of interest.

## Publisher’s Note

All claims expressed in this article are solely those of the authors and do not necessarily represent those of their affiliated organizations, or those of the publisher, the editors and the reviewers. Any product that may be evaluated in this article, or claim that may be made by its manufacturer, is not guaranteed or endorsed by the publisher.
