# Supplementary material for: Osteopontin N-Terminal Function in an Abdominal Aortic Aneurysm From Apolipoprotein E-Deficient Mice
Source: Front Cell Dev Biol. 2021 Aug 12;9:681790. doi: 10.3389/fcell.2021.681790 (PMC8397420; doi:10.3389/fcell.2021.681790)
Supplement: Supplementary file 1 [file Data_Sheet_1.pdf]

Supplemental material

Figure 1S：Mapping of antibody sequence of this study.

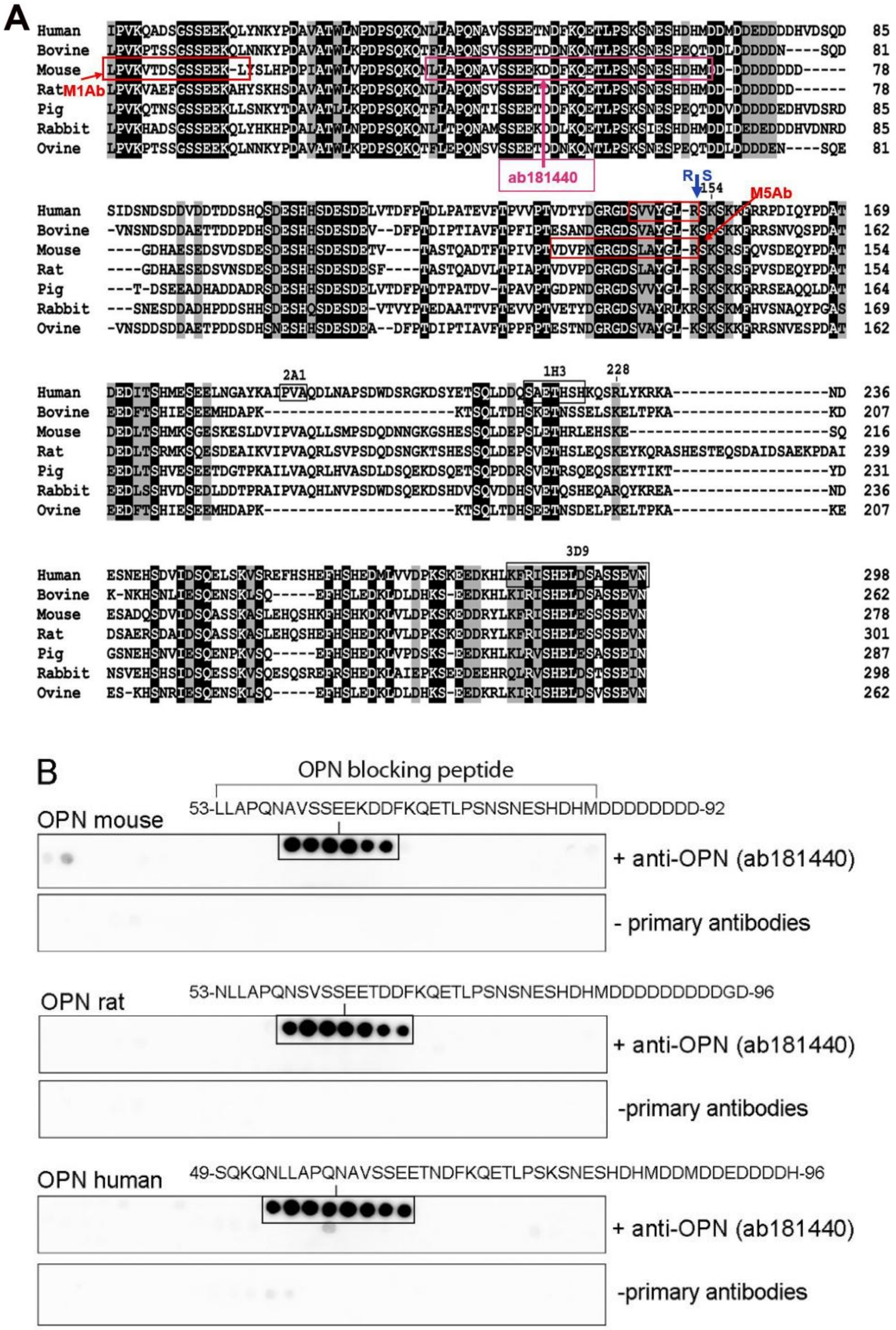

Figure 1S: Mapping of antibody sequence of this study. **(A)** The mammalian OPN sequence diagram was sited in figure 1 of reference "PMID 22179617", on the basis of which the sequences of the antibody used in this study were shown. Identical and/or similar amino acid residues are aligned to the maximum extent possible, with identical amino acids marked in black and structurally similar amino acids in gray. The thrombin cleavage site is indicated in the mouse, rat and human OPN sequences in A (blue arrow). M5 peptide (CVDVPNGRGDSLAYGLRS) and M1 peptide (LPVKVTDSGSSEEKL) ware boxed in red. Sequence of the anti-OPN-N (ab181440) is shown in upper panel in A (pink arrow). **(B)** Anti-OPN-N were overlaid arrays of immobilized overlapping 20-mer peptides covering the mouse, rat or human OPN protein sequence (PMID **32000579)**.

Figure 2S： Negative controls for IHC and IF staining were used as supplementary data.

Figure 2S-A:

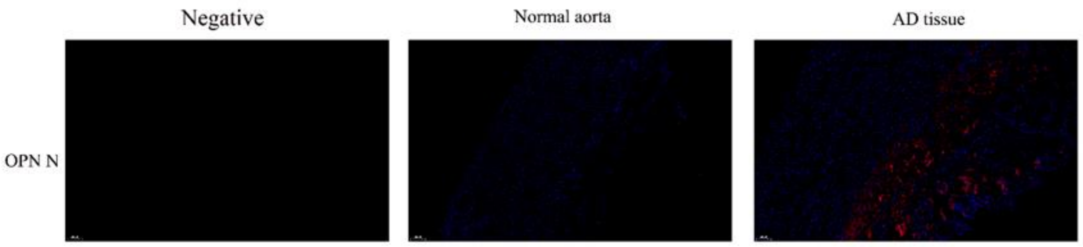

Figure 2S-A: Representative immunofluorescence staining for OPN-N in AD tissue and normal aorta (Scar bar 200 μm; DAPI staining of nuclei:blue; OPN-N staining: red).

Figure 2S-B:

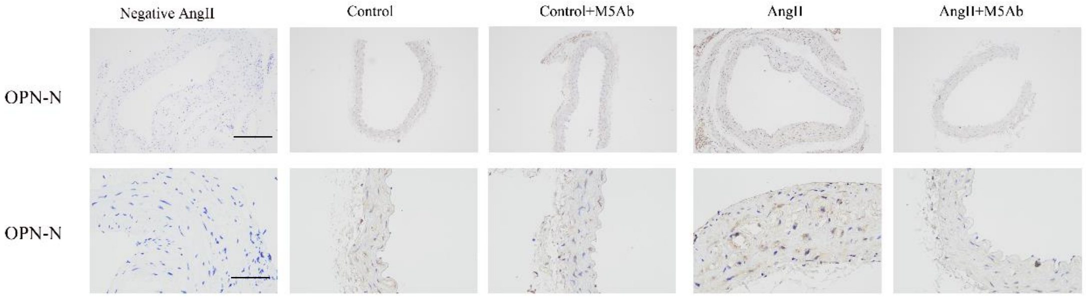

Figure 2S-B: Representative images of aortic immunohistochemistry staining to detect OPN-N expression in mice. 100μm,above; 50μm,below.

Figure 2S-C:

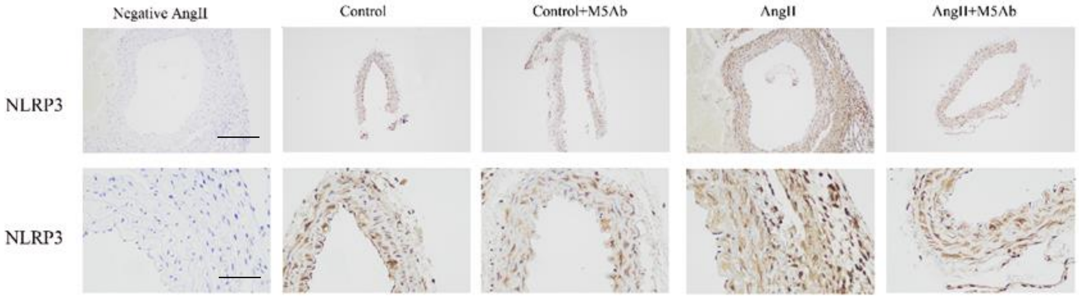

Figure 2S-C: Immunohistochemistry staining of NLRP3 in mice under different treatments. 100μm,above; 50μm,below.

Figure 3S：The species matched irrelevant control antibodies should be used.

The M1Ab is a polyclonal antibody prepared against the M1 peptide (LPVKVTDSGSSEEKL) of the amino terminus of OPN, which does not block the functions of OPN-N. M1Ab has been used as control antibody for M5Ab in many studies (PMID 15485631; PMID 18836077; PMID 30350863). In the pre-experimental stage, we had choosed IgG and M1Ab as M5Ab negative control antibodies, and designed four groups with five mice in each group: AngII group, AngII+IgG group, AngII+ M1Ab group, AngII+ M5Ab group. The IgG, M1Ab and M5Ab (HuaAn Biotechnology, Hangzhou, China) were administered to mice tails intravenously (400 μg each injection, two injections per week for the final 2 weeks after implantation of Alzet osmotic minipumps). The methods of animal feeding and modeling were exactly the same as those described in the manuscript of this study. The reagents and IHC/WB antibodies used were the same as those used in this study.

We found that degradation of the elastic fibers and collagen deposition was significantly decreased in AngII+ M5Ab mice compared with other treatments (Figure 3S-A). Interestingly, administration of the M5Ab significantly decreased the expression of OPN-N in ApoE-/- mice (Figure 3S-A, 3S-B). In contrast, the administration of control antibody (IgG or M1Ab) did not attenuated the degradation of elastic fibers and collagen deposition, and did not reduced the expression of OPN-N, α9β1, NLRP3, NF-κB (Figure 3S-A, 3S-B). Since antibody itself may be effective to reduce the inflammation responses, this study indicating that M5Ab weakened the Ang II-induced AAA by reducing OPN-N expression, independent of the antibody themself.

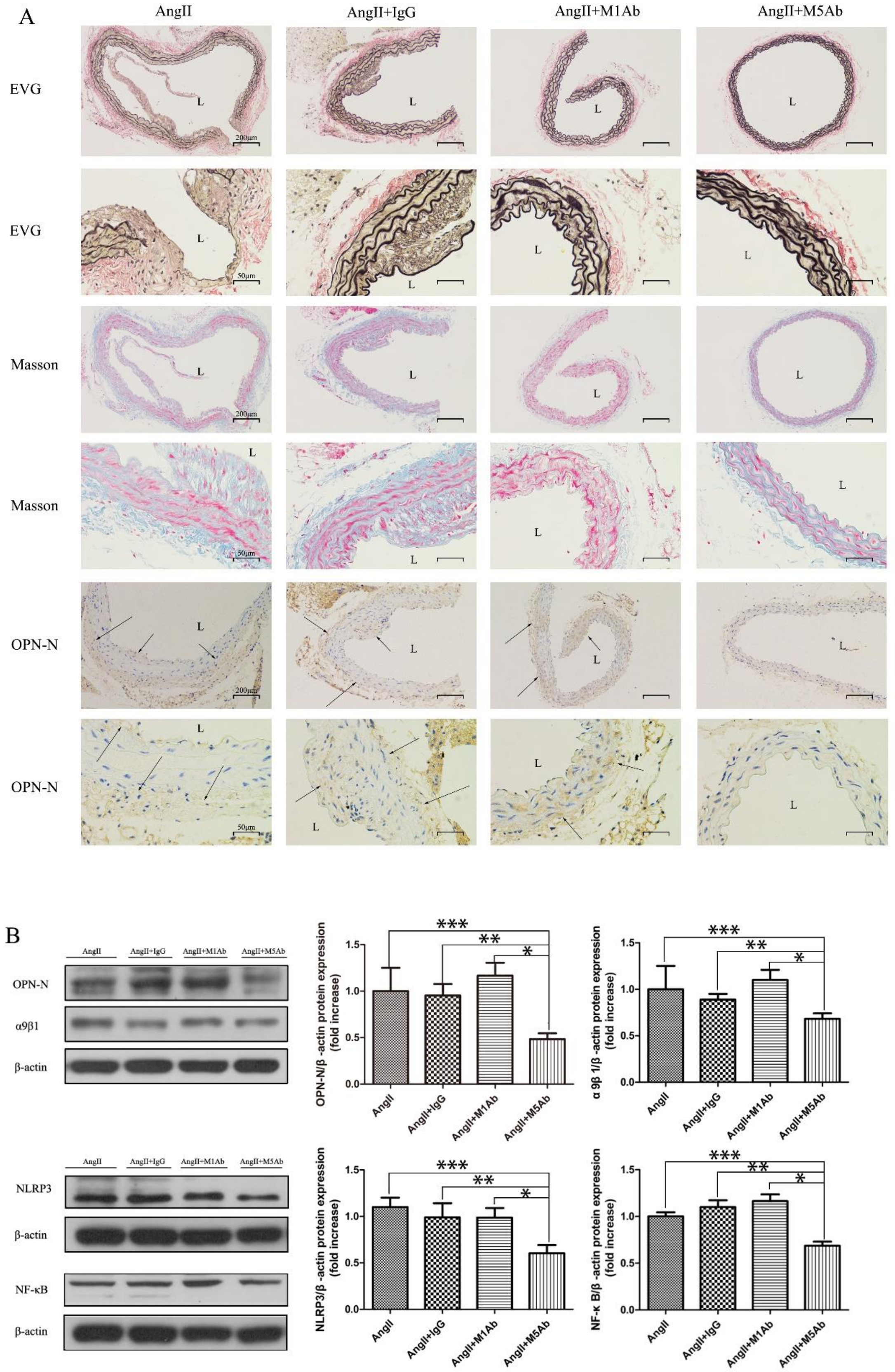

Figure 3S-A: Administration of M5Ab attenuated the Ang II-induced AAA. **(A)** AngII-induced AAA mice were treated with under different treatments, and degradation of the elastic fibers (EVG staining, black), collagen depositions (Masson's trichrome staining, blue), and OPN-N

expression (immunohistochemistry staining, yellow with black arrows) were evaluated in ApoE-/- mice (Scar bar 200 μm or 50 μm). **(B)** Western blotting analyses of the protein levels of OPN-N, α9β1, NLRP3, NF-κB in mice, and quantification of these proteins expression. β-actin was used as an internal control. Data are expressed as means ± SEM; n = 3 per group. *$P < 0.05$, Ang II + M1Ab vs the Ang II + M5Ab group; **$P < 0.05$, Ang II + IgG vs the Ang II + M5Ab group; ***$P < 0.05$, Ang II vs the Ang II + M5Ab group;
